# Mobile Phone Use “on the Road”: A Self-Report Study on Young Drivers

**DOI:** 10.3389/fpsyg.2021.620653

**Published:** 2021-08-16

**Authors:** Angelo Fraschetti, Pierluigi Cordellieri, Giulia Lausi, Emanuela Mari, Elena Paoli, Jessica Burrai, Alessandro Quaglieri, Michela Baldi, Alessandra Pizzo, Anna Maria Giannini

**Affiliations:** Department of Psychology, Sapienza University of Rome, Rome, Italy

**Keywords:** multitasking, road safety, teenagers, driving, mobile phone use, gender differences, SEM modeling

## Abstract

**Background:**

Extensive research showed that multitasking negatively affects driving performance. Multitasking activities can range from talking and texting to listening to music; particularly among young drivers, multitasking behavior is caused mainly from mobile phone use while driving which is one of the main causes of road accidents.

**Objective:**

The main purpose of this study was to investigate whether some variables (e.g., Sensation-Seeking, preferences of Multitasking) could affect mobile phone use while driving in young drivers and whether any gender differences were present among the examined variables.

**Setting and participants:**

The sample consists of 424 Italian students (56% males) with an age range of 18–21 years. A self-report questionnaire was specifically developed to assess variables such as: Attitude toward Multitasking, Perceived Self-efficacy in Multitasking, Accident Risk Perception, General Multitasking Habits, and Sensation Seeking.

**Results:**

Through SEM modeling, we found the attitude to multitasking while driving to be largely explained by the considered variables. Using multigroup analysis (MGSEM), the model we developed appears to be suitable for explaining the behaviors of both male and female young drivers. Furthermore, data comparison showed that females were more likely to risk perception toward multitasking, and risk perception when using a mobile phone while driving, while males obtained higher mean scores in Sensation Seeking, Perceived Self-Efficacy in Multitasking, and in Multitasking caused by mobile phone use while driving.

**Conclusion:**

Our research showed how some variables may influence the inclination of some subjects to engage in multitasking while driving. Furthermore, we discussed the importance of considering these variables in the implementation of effective road safety education projects on driving multitasking.

## Introduction

According to the World Health Organization’s (WHO) global status report in 2018, road accidents are the eighth leading cause of death worldwide, with approximately 1.35 million deaths and up to 50 million injuries per year (World Health Organization (WHO), 2018). In Italy, in 2019, 3,173 people lost their lives in road accidents, and 241,384 were injured (Istat, 2019). Moreover, in Italy 94.7% of road accidents are caused by driver or pedestrian misconduct, such as distracted or hesitant driving, lack of respect for traffic lights or priorities and speeding (Istat, 2019).

Human errors in traffic—caused by mental workload—and transgressions of safety rules and traffic codes, have been considered a crucial factor in road accidents (Brookhuis and De Waard, 2010; Paxion et al., 2013); being the primary cause of road accidents (93%, i.e., driving ability, excessive speed), followed by different environmental factors (34%; i.e., road signs, visibility and weather conditions) and vehicle-related factors (12%; i.e., service or car design) (Fazel and Zad, 2007).

Among the main human errors leading to traffic accidents, the distraction of the driver plays a key role, primarily due to multitasking activities while driving.

Driving per se involves several simple and more complex tasks that also include decision-making processes; however, including the availability of multimedia systems and other distractions out of the vehicle, driving becomes a challenging “multitasking environment” (Salvucci and Taatgen, 2008).

Sometimes individuals perform the secondary task while driving, producing both visual and motoring distractions and inattention. Extensive research has shown that multitasking while driving has a negative impact on driving performance: it increases reaction times (Gershon et al., 2009; Atchley and Chan, 2011; He et al., 2014; Nijboer et al., 2016; Jokinen et al., 2020; Kim et al., 2020). One common multitasking while driving is definitely the use of mobile phones (e.g., handheld or hands-free texting) with consequent negative effects on an individual’s performance (Salvucci and Macuga, 2002; Strayer et al., 2003; Patten et al., 2004; Horrey and Wickens, 2006; Hosking et al., 2006; Drews et al., 2008; Cooper et al., 2011; Yager et al., 2012; Hill et al., 2021; Keffane, 2021; Sullman et al., 2021; Truelove et al., 2021; Vollrath et al., 2021). Several studies have investigated brain activity during careless driving (Schweizer et al., 2013; Karthaus et al., 2018). Recently, in a systematic review, Palmiero et al. (2019) showed how the brain activations associated with driving decrease when a secondary task is added.

The risk of driving accidents is four times higher when drivers are using their mobile phones (McEvoy et al., 2005). In fact, use of the mobile phone while driving induces a form of inattentional blindness, meaning that drivers fail to notice information in their line of sight (Strayer et al., 2011).

Previous research investigated possible differences between men and women in mobile phone use while driving, as well as differences between young and older adults. However, most studies have shown that differences defining these groups are wildly inconsistent (Watson and Strayer, 2010; Mäntylä, 2013; Stoet et al., 2013; Strayer et al., 2013; Todorov et al., 2014). Moreover, each individual, regardless of gender, has very different skills in specific tasks that would not result from innate differences in multitasking but rather from different individual skills and abilities (Salvucci and Taatgen, 2008). With respect to the role of age on mobile phone use while driving, the same systematic review showed that younger drivers are more likely to use a mobile phone while driving than older drivers (Huemer et al., 2018). Therefore, it seems important to study which variables may influence mobile phone use in young drivers and to investigate the psychological aspects of distracted driving to provide useful information for road safety training.

### Objectives and Hypotheses

Based on the above premises, the first aim of the study was to assess the role of some variables (e.g., Sensation-Seeking, preferences of Multi-tasking) in distracted driving behaviors due to mobile phones use. By using a SEM model, we investigated variables that could predict mobile phone use among young adults.

Secondly, we evaluated the gender differences in the use of mobile phones while driving. This issue is still unclear. In a systematic review Huemer et al. (2018) showed contradictory results, indeed, of the 51 papers considered, 24% reported that females have higher mobile phone use, 30% found that males have higher mobile phone use while the 46% of the studies found no gender difference.

## The Study

The approach to empirical research adopted for this study starts from four psychological factors that are known to have a role in general multitasking and in distracted driving in young people. These factors are described below.

### Attitudes Toward Multitasking: Polychronicity

As already mentioned, multitasking is a complex dimension in which several factors are involved, including Polychronicity. This term refers to the preference of a subject to engage in several activities at the same time rather than individual sequential ones with the confidence of being able to correctly perform several tasks at the same time. Slocombe and Bluedorn (1999) describe Polychronicity as a stable trait of personality, and other studies have confirmed the presence of this specific trait (Poposki and Oswald, 2010). Bluedorn et al. (1999) developed an inventory to measure the tendency (propensity, disposition) of subjects towards Polychronicity (Inventory of Polychronic Values, IPV). Research suggests that among young drivers, a high level of commitment to mobile phone use is found during the driving experience. This affects the safety of the driver and is related to the frequency of accidents during these actions (Terry and Terry, 2015). In a recent study, gender differences were found with respect to the Polychronicity trait, where women show higher scores than men, clearly demonstrating a higher personal preference by women to perform several tasks at once. Furthermore, women reported spending more time multitasking and considered multitasking to be more important in everyday life than men. Finally, Polychronicity seems to correlate positively with self-rated multitasking abilities (Szameitat and Hayati, 2019).

### Self-Efficacy in Multitasking

The relevance of realistic perception of one’s skills has also been investigated in traffic psychology, demonstrating that most drivers tend to overestimate their skills (Matthews and Moran, 1986; Hatakka et al., 2002; Yanuvianti et al., 2020). In particular, self-efficacy has been defined as judgment of the ability to organize and perform actions to achieve specific goals (Bandura, 1997).

Self-efficacy within the driving context has been associated with a more frequent shift of attention to concomitant processes (e.g., mobile phone use while driving) (Wang, 2016). A study by Schlehofer et al. (2010) suggests that self-efficacy plays a compensatory role with respect to the risks related to driving, providing for more frequent use of mobile phones while driving. In addition, a previous study examined the mediator role of self-efficacy while driving in relation to specific personality traits (Wang et al., 2018; Zhang et al., 2020).

Literature on gender differences in self-efficacy remains unclear; in fact, some studies of self-rated driving ability did not examine gender (Freund et al., 2005), while others did not report gender differences (Marottoli and Richardson, 1998; Blanchard and Myers, 2010). In contrast, an interesting study by Ackerman et al. (2011) investigating indicators of self-efficacy in driving across a 3-month interval reported that gender was predictive of self-rated driving ability. Men assessed their driving ability more highly at follow-up compared to the baseline. In addition, a study by Nasvadi (2007) reports that compared to women, men report greater comfort in their driving ability and are more likely to report enhanced driving ability after a traffic education program. The authors suggest that this overestimation of driving skills may be due to use of a strategy to maintain a positive self-image. Both results in these studies have been obtained in a sample of older adults, so it could be interesting to investigate the possible presence of gender differences, even in a younger population.

### Risk Perception

Risk perception is an important predictor in the assumption of road risk behavior among the psychological determinants analyzed by traffic psychology (Brown and Groeger, 1988; Horwarth, 1988). In particular, Wilde (1994) has shown that driving behavior is influenced by subjective risk perception. It was previously observed that those who perceive high levels of risk engage in behaviors such as reduced speed increased attention while driving, as well as compliance with traffic rules, in contrast to those who perceive low levels of risk (Slovic et al., 2005; Cordellieri et al., 2019; Song et al., 2021).

Recent studies have shown that drivers perceive the use of a mobile phone while driving as a highly risky behavior (Zhang et al., 2020) and that risk perception can influence the decision to use a mobile phone while driving (Przepiorka et al., 2018).

Age and gender also seem to play an important role in risk perception and willingness to take a risk in traffic. Bragg and Finn (1982) pointed out that low risk perception by young people may depend on their belief in being able to control dangerous situations, overestimating their skills (Matthews and Moran, 1986; Taubman-Ben Ari et al., 2004; Delhomme et al., 2009; Lucidi et al., 2010; Guggenheim et al., 2020) and underestimating the serious consequences of implementing dangerous behavior (Lucidi et al., 2019). In particular, Glendon et al. (1996) showed that young male drivers tend to underestimate their personal perception of risk and overestimate their competence compared to women. Recent studies have addressed gender differences in different risk profiles, demonstrating a higher risk tendency among adolescent males, although the level of risk perception was found to be the same in both genders (Cordellieri et al., 2016, 2019).

### Sensation Seeking

Sensation seeking is a component of the personality that drives individuals to search for new and intense experiences (Zuckerman, 1979). Traditionally, some personality traits have been studied in relation to their ability to affect risky driving behavior (Ulleberg and Rundmo, 2003; Dahlen et al., 2005; Schwebel et al., 2006; Tao et al., 2017; Al-Tit, 2020). Recent research found that, among these personality factors, sensation seeking is a predictor of risky driving behavior (Lemarié et al., 2019; Jamt et al., 2020; Qu et al., 2020).

According to Adan et al. (2016), males exhibit higher scores than females with respect to sensation seeking; nevertheless, higher sensation seeking traits that are associated with risky behaviors are more problematic in females (Navas et al., 2019).

A recent meta-analysis (Zhang et al., 2019) found that sensation seeking was not only associated with risky driving, but also with other behaviors, such aggressive driving, error and other misconducts. This confirms the important role that sensation seeking plays in driving behaviors: drivers with higher levels of sensation seeking are more likely to commit errors and to be exposed to a higher risk of accidents.

Starting from the scientific literature on multitasking and distracted driving described above, we selected variables to use in our model on mobile phone use while driving. To the factors listed above, we added then a fifth factor: engaging in multitasking behavior. Our hypothesis is that subjects who are more prone to engage in general multitasking behavior (i.e. studying while listening to music, playing videogames while talking to the mobile phone) are also more prone to use their mobile phones while driving.

## Materials and Methods

### Participants

For this cross-sectional study, a total sample of *N* = 424 Italian high school students (male = 56.1%) aged from 18 to 21 years (mean age = 18.38, SD = 1.029) was recruited, distributed across different Italian regions (38.9% Northern, 23,1% Central, and 38% Southern) with different driving experiences. All participants were car drivers. None of the participants reported to have been involved in severe car crashes. Participants were recruited from schools previously agreeing to take part in an educational project, the surveys were administered in classroom with the authorization and cooperation of the participants, and educational staff (teachers, project trainers) were involved in road safety training. Participants from schools were then randomly selected from each institute, and pupils agreed or disagreed to participate. This study was approved by the Ethics Review Board of the Department of Psychology, “La Sapienza” University of Rome (IRB 2414/2019); participants were informed of the aims and purpose of the study, as well as their participation rights (e.g., confidentiality of responses, allowance to leave the study at any point without any consequences), in advance of data collection. Thus, written informed consent was obtained from all participants.

### Materials and Procedures

For this study, a paper-and-pencil questionnaire was created, consisting of multiple sections. First, a short summary of demographic data (i.e., age, gender, driving experiences) was created; the following sections consist of different measures, explored below:

#### Scale A. Adolescent Multitasking Preference Inventory (AMPI: Measure of Individual Differences Polychronicity).

This scale was derived from the Multitasking Preference Inventory (MPI; Poposki and Oswald, 2010), which has become one of the most widely used scales for assessing self-reported multitasking preference (e.g., “When I have a task to complete, I like to break it up by switching to other tasks intermittently”). Eleven items completed the scale, and all the answers were on a five-point Likert scale ranging from “strongly disagree” (1) to “strongly agree” (5).

#### Scale B. Perceived Self-Efficacy in Multitasking

This scale measures individuals’ beliefs about one’s ability to multitask. Respondents indicated how much they perceived themselves capable of performing certain actions at the same time (e.g., “studying and answering messages on the phone”). Eight items completed the scale, and all responses were on a five-point Likert scale ranging from “very low” (1) to “very high” (5).

#### Scale C. Accident Risk Perception Due to Multitasking

Respondents indicated how much they perceived the risk of certain multitasking behaviors while driving. Ten items were used to measure this risk perception. Items referred to risk conditions with a mobile phone (e.g., “type a message on your mobile phone while driving”) and risk situation without a mobile phone (e.g., “eating in the car: sandwich, snacks, etc.”). Rating was performed using a five-point Likert scale from “not at all risky” (1) to “very risky” (5).

#### Scale D. Sensation Seeking

Sensation seeking is a personality trait defined by the degree to which an individual seeks novel and highly stimulating activities and experiences. We used five items from the “NEO Personality Inventory” (Costa and McCrae, 2008) to measure this personality aspect (e.g., “I often wish exciting things”). Respondents were required to respond on a six-point Likert scale from “strongly disagree” (0) to “strongly agree” (5).

#### Scale E. Multitasking Behavior (MB)

As a measure of multitasking behavior had not yet been developed, we created a new scale. Seventeen questions aimed to measure the frequency of multitasking behavior in different situations were designed. Respondents were required to indicate answers on a five-point Likert scale from “Never” (1) to “Very often” (5) how often they have engaged in multitasking behaviors in general situations (e.g., “listening to background music while I’m studying”), as a pedestrian (e.g., “crossing the street looking at messages on your mobile phone”) and while driving a vehicle (e.g., “read or write while you’re driving”).

### Statistical Analysis (Data Processing)

Before running the analysis, data were controlled for missing data and outliers examining box plots (e.g., we did not consider students over the age of 21). No missing data were found. Normality, Linearity, and Homoscedasticity were also assessed.

First, data from the different scales were separately submitted to exploratory factor analysis using the Principal Axis method and the oblique Oblimin rotation. Factor scores were then computed through the regression method for each factor and used for further statistical analyses.

After performing basic descriptive analyses, bivariate correlation (Pearson) analysis was performed to establish potential relationships among the considered variables in the case study of this sample of Italian students.

Furthermore, associations among different factor scores concerning multitasking were tested using path analysis [structural equation modeling (SEM) with maximum likelihood estimations] with the following significance parameters: *p* < 0.05, *p* < 0.01, and *p* < 0.001.

To examine possible gender differences, factor scores for each Scale were separately submitted to multivariate analysis (MANOVA), with gender (female and male) as independent variables and factors score as dependent variable. A separate ANOVA was conducted for each dependent variable, with each ANOVA using the Bonferroni correction for alpha inflation due to multiple testing (Bonferroni corrected α = α/K; K = Number of tests).

We used Independent-sample *t*-test when the Scale had only a single factor.

Finally, the same model was tested for the second time, using a gender-based multi-group analysis (MGSEM with MLA) with differential criteria-significance levels of *p* < 0.05, *p* < 0.01, and *p* < 0.001. All statistical analyses were performed using IBM SPSS (Statistical Package for Social Sciences) version 24.0, and IBM SPSS AMOS, version 22.0, was principally used for conducting structural analyses.

## Results

### Exploratory Factor Analysis

#### Scale A: Adolescent Multitasking Preference (Attitudes Toward Multitasking)

Data from the Adolescent Multitasking Preference Inventory were submitted to exploratory factor analysis (Principal Axis method, Oblimin rotation). Measures of sampling adequacy (Kaiser-Meyer-Olkin = 0.739) and factorability of the correlation matrix [Bartlett’s test of sphericity χ^2^ (36) = 587.983, *p* < 0.001] were both adequate. The scree-test yielded a three-factor solution accounting for 57.12% of the total variance. The first factor labeled “Carelessness,” accounted for 30.52% of the common variance and referred to the positive attitude toward lack of awareness during a behavior that can result in unintentional consequences. Items such as “I like to immerse myself in my fantasies while listening to the teacher explain” loaded on this factor.

The second factor, labeled “Multitasking Preference (Polychronicity),” accounted for 14.44% of the common variance and referred to the positive attitude toward performing one or more tasks concurrently, in contrast to performing only one single task at a time. Items such as “One day I’d like to do a job where I have to do several things simultaneously” were included here. This factor showed a slight positive correlation with the first factor (0.32). The third factor, called “Concentration,” represented 12.5% of the common variance and referred to a positive attitude toward attentional processes that involved the ability to concentrate on the task, while ignoring multitasking. Elements such as “I prefer not to be interrupted while I am busy studying” loaded this factor. The third factor negatively correlated with the first (−0.39) and the second (−0.21) factor.

#### Scale B: Perceived Self-Efficacy in Multitasking

Exploratory factorial analysis (Principal Axis method, Oblimin rotation) on the scale of Perceived self-efficacy in Multitasking yielded a solution to one factor. Measures of sampling adequacy (Kaiser-Meyer-Olkin = 0.713), and factorability of the correlation matrix [Bartlett’s test of sphericity χ^2^ (15) = 205.745, *p* < 0.001] were both adequate. The one-factor solution explained 50.22% of the variance. This factor referred to the individual’s judgment of his or her ability to perform certain actions successfully at the same time. Items such as “Studying and texting on the phone” loaded on this factor.

#### Scale C: Accident Risk Perception Due to Multitasking

Data from the Accident risk perception due to multitasking were submitted to exploratory factor analysis (Principal Axis method, Oblimin rotation). Measures of sampling adequacy (Kaiser-Meyer-Olkin = 0.835) and factorability of the correlation matrix [Bartlett’s test of sphericity χ^2^ (36) = 1665.53, *p* < 0.001] were both adequate. The scree-test yielded a two-factor solution accounting for 61.58% of the total variance. The first factor labeled “Risk perceptions when using a mobile phone while driving,” accounted for 47.85% of the common variance. Items such as “Taking a selfie while driving” loaded on this factor. Higher scores in this factor correspond to a greater perception of risk. The second factor labeled “Risk perception about multitasking without using a mobile phone,” accounts for 13.73% of the common variance and referred to risk perception in multitasking behavior but not while driving. Items such as “Arguing passionately with a passenger” loaded on this factor. This factor positively correlated with the first one (0.49).

#### Scale D: Sensation Seeking

Exploratory factorial analysis (Principal Axis method, Oblimin rotation) on the scale of Sensation Seeking yielded a solution to one factor. Measure’s sampling adequacy (Kaiser-Meyer-Olkin = 0.730), and factorability of the correlation matrix [Bartlett’s test of sphericity χ^2^ (6) = 330.286, *p* < 0.001] were both adequate. The one-factor solution explained 54.87% of the variance. This factor referred to the tendency to seek intense sensations. According to Zuckerman (1994), sensation seeking (SS) is a trait defined by the seeking of varied, novel, complex, and intense sensations and experiences and the willingness to take physical, social, legal, and financial risks for the sake of such experiences. Items such as “I like to be where the action is” loaded on this factor.

#### Scale E: Multitasking Behavior

In addition, data from the Imagined Multitasking Behavior Scale were submitted to exploratory factor analysis (Principal Axis method, Oblimin rotation). Measures of sampling adequacy (Kaiser-Meyer-Olkin = 0.691) and factorability of the correlation matrix [Bartlett’s test of sphericity χ^2^ (21) = 501.39, *p* < 0.001] were both adequate. The scree-test yielded a two-factor solution accounting for 52.63% of the total variance. The first factor labeled “Multitasking in driving using the phone,” accounted for 33.26% of the common variance and referred to drivers who frequently take on driving and multitasking. Items such as “Driving and talking on your mobile phone” loaded on this factor. The second factor, labeled “General Multitasking Behavior,” accounted for 19.36% of the common variance and especially referred to multitasking behavior in different contexts, not in driving situation. Items such as “Watching TV while I’m doing my homework” loaded on this factor. This factor positively correlated with the first one (0.33).

### Explaining Multitasking in Driving While Using the Phone: SEM Modeling

The bivariate correlation analysis (Table 1) allowed us to establish statistically significant measures of association among study variables related to Multitasking in driving in young Italian students.

**TABLE 1:** Pearson bivariate correlations of the investigated variables.

	Scale		Variable	Bivariate correlations (2-tailed)								
				1	2	3	4	5	6	7	8	9
A	Attitude toward multitasking Adolescent Multitasking Preference Inventory (AMPI)	1	Carelessness	1	0.304***	−0.348***	0.329***	−0.223***	−0.141***	0.255***	0.304***	0.478***
		2	Multitasking Preference (Polychronicity)		1	−0.337***	0.275***	−0.117*	−0.102*	0.277***	0.291***	0.283***
		3	Concentration			1	−0.335***	0.207***	0.179***	−0.184***	−0.303***	−0.349***
B	Cognitive control	4	Perceived self−efficacy in Multitasking				1	−0.081	−0.161*	0.273***	0.274***	0.581***
C	Accident risk perception due to multitasking	5	Risk perceptions when using a mobile phone while driving					1	0.491***	−0.176***	−0.368***	−0.147***
		6	Risk perception about multitasking without using a mobile phone						1	−0.198***	−0.263***	−0.128**
D	Personality trait	7	Sensation-Seeking							1	0.361***	0.328***
E	Multitasking behavior	8	Multitasking in driving using the phone								1	0.344***
		9	General Multitasking Behavior									1

N = 424. *p < 0.05. **p < 0.01. ***p < 0.001.

Regarding some important correlations found directly among the variables, it was found that Multitasking in Driving Using the Phone were significantly associated with Concentration [−], Carelessness [+], Multitasking Preference [+], Perceived Self-Efficacy in Multitasking [+], Risk Perception when using a mobile phone while driving [−], Risk perception about multitasking without using a mobile phone [−], Sensation Seeking [+] and General Multitasking Behavior [+].

Based on the theoretical roots presented in the introduction, the effect of the variables related to Multitasking in Driving, evaluated through the questionnaire administered to a sample of Italian students with SEM (structural equation modeling) approach, was examined. Using SPSS AMOS path analyses, the hypothesized structural model was adjusted to fit the data, while considering the parameters of the full sample, which was accomplished with the minimum sample size as suggested by the by the literature.

**FIGURE 1:**
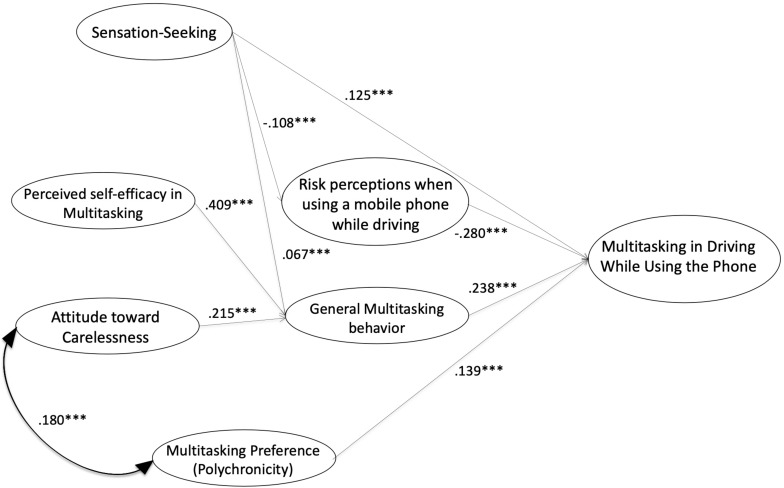
Standardized path coefficients of structural model for Multitasking in driving using the phone. ^∗∗∗^*p* < 0.001.

A baseline (a priori) model did not fit the data well [χ^2^ (20) = 263.3, *p* < 0.001; Normed Fit Index (NFI) = 0.684; Comparative Fit Index (CFI) = 0.691; Root Mean Square Error of Approximation (RMSEA) = 0.170] and needed to be adjusted. Therefore, several modifications were made. First, non-significant and very low paths were set to zero, eliminating some variables. Specifically, Attitude toward Concentration and Risk perception about multitasking without using a mobile phone were excluded in the first significant model. Secondly, a very large Modification Index was used indicated a relevant relationship between the independent variables and risky behaviors. These modifications made the model parsimonious, giving a model fit that was adequate. The resulting structural equation model was more parsimonious and reported better fit coefficients [χ^2^ (7) = 20.19, *p* < 0.05; NFI = 0.967; CFI = 0.976; RMSEA = 0.052; Minimum indicating a good Discrepancy/Degrees of Freedom (CMIN/DF) = 2.885], all of which were acceptable and indicated a good model fit and are presented in Figure 1.

In brief, the standardized path coefficients (see Table 2 and values next to solid lines in Figure 1) of the model showed positive associations between Polychronicity (β = 0.139^∗∗∗^), General Multitasking Behavior (β = 0.238^∗∗∗^), and Sensation-Seeking (β = 0.125^∗∗∗^) with Multitasking in Driving Using the Phone. Differently, Risk perceptions when using a mobile phone were a good predictor of negative association (β = −0.280^∗∗∗^). Positive Attitude toward Carelessness (β = 0.215^∗∗∗^), Perceived self-efficacy in Multitasking (β = 0.409^∗∗∗^), and Sensation Seeking (β = 0.067^∗∗∗^) showed links with General Multitasking Behavior. Lastly, Sensation Seeking is a negative predictor of the Risk perceptions when using a mobile phone while driving (β = −0.108^∗∗∗^).

**TABLE 2:** Structural Equation Model (SEM) for predicting the multitasking in driving using the phone.

Dependent variable		Independent variable	Std. estimate^a^	SE^b^	CR^c^	*p* ^d^
Multitasking in driving using the phone	←	Multitasking Preference (Polychronicity)	0.139	0.041	3.379	0.001
Multitasking in driving using the phone	←	Risk perceptions when using a mobile phone while driving	−0.280	0.040	−7.043	0.001
Multitasking in driving using the phone	←	General Multitasking Behavior	0.238	0.050	4.766	0.001
Multitasking in driving using the phone	←	Sensation-Seeking	0.125	0.027	4.692	0.001
Multitasking in driving using the phone	←	Risk perception about multitasking without using a mobile phone*	−0.066	0.038	−1.762	n.s.
General Multitasking Behavior	←	Attitude toward Carelessness	0.215	0.029	7.460	0.001
General Multitasking Behavior	←	Sensation-Seeking	0.067	0.020	−3.313	0.001
General Multitasking Behavior	←	Perceived self-efficacy in Multitasking	0.409	0.037	11.190	0.001
General Multitasking Behavior	←	Attitude toward Concentration*	−0.05	0.026	−2.216	n.s.
Risk perceptions when using a mobile phone while driving	←	Sensation-Seeking	−0.108	0.030	−3.617	0.001

^a^SPC, Standardized Path Coefficients (can be interpreted as linear regression weights). ^b^SE, Standard Error. ^c^CR, Critical Ratio. ^d^p-values. *These variables were excluded from the model.

As mentioned, positive attitude toward Concentration and Risk perception about multitasking without using a mobile phone were not significant.

### Gender Differences

#### Attitudes Toward Multitasking (Scale A)

**FIGURE 2:**
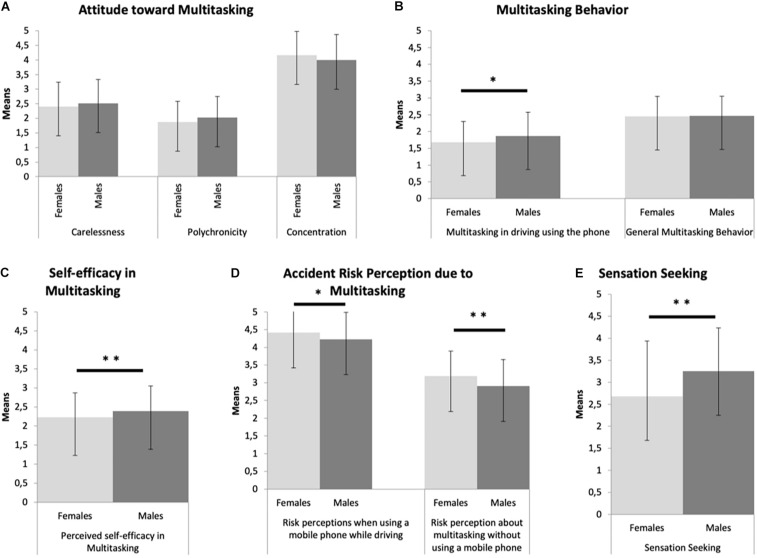
Mean factor scores for the five dimensions [**(A)** Attitude toward Multitasking; **(B)** Multitasking Behavior; **(C)** Self-efficacy in Multitasking; **(D)** Accident Risk Perception due to Multitasking; **(E)** Sensation-Seeking] represented for gender. ^∗^*p* < 0.05, ^∗∗^*p* < 0.01.

In order to verify the presence of gender differences (Figure 2), we used a multivariate analysis of variance (MANOVA) with gender (female and male) as a between-subjects factor, and the overall scores of each dimension of Attitudes toward Multitasking (i.e., Carelessness, Multitasking preference, and Concentration) as dependent variables. With respect to the Attitude toward Multitasking there was no statistically significant difference of gender (Wilk’s Λ = 0.984, *F*_(3,410)_ = 2.19, *p* = 0.089).

#### Accident Risk Perception Due to Multitasking (Scale B)

The was a statistically significant difference for gender (Wilk’s Λ = 0.963, *F*_(2,414)_ = 7.91, *p* = 0.000, *η^2^_*p*_* = 0.037). A separate ANOVA was conducted for each dependent variable, with each ANOVA evaluated at an alpha of 0.008 (Limit set by Bonferroni correction: α/K; K = Number of tests). There was a significant difference between males and females for Risk perceptions when using a mobile phone while driving [*F*_(1,415)_ = 7.043, *p* = 0.0079, *η^2^_*p*_* = 0.017), the females’ group showed higher mean (*M* = 4.42, SD = 0.636) than males’ group (*M* = 4.23, SD = 0.423). There was also a statistically significant difference between males and females with respect to the Risk perception about multitasking without using a mobile phone [*F*_(1,415)_ = 15.045, *p* = 0.000, *η^2^_*p*_* = 0.035), in which the mean of the females’ group was higher (*M* = 3.19, SD = 0.707) than in males’ group (*M* = 2.91, SD = 0.746).

#### Multitasking Behavior (Scale E)

The MANOVA revealed a statistically significant gender effect [Wilk’s Λ = .980, *F*_(2,405)_ = 4.16, *p* = 0.016, *η^2^_*p*_* = 0.020]. A separate ANOVA was conducted for each dependent variable, with each ANOVA evaluated at an alpha of 0.008 (Bonferroni correction). The Multitasking in driving while using the phone dimension, showed a statistically significant difference between males and females [*F*_(1,406)_ = 7.778, *p* = 0.006, *η^2^_*p*_* = 0.019), the males’ group reported higher mean (*M* = 1.86, SD = 0.707) compared to the females’ group (*M* = 1.68, SD = 0.617). There was no statistically significant result of General Multitasking Behavior [*F*_(1,406)_ = 0.070, *p* = 0.791].

Independent-sample *t*-test analyses were conducted on Perceived self-efficacy in Multitasking (Scale B) and on Sensation Seeking (Scale D). The Perceived self-efficacy in Multitasking scale showed a statistically significant higher mean in the males’ group (*M* = 2.39, SD = 0.661) than in females’ group (*M* = 2.23, SD = 0.640), *t*_(1, 414)_ = 2.440; *p* < 0.05, *d* = 0.241. With respect to the Sensation Seeking (Scale D) there was a significant difference between males and females *t*_(1, 336)_ = 5.217; *p* < 0.001, *d* = 0.514). Leven’s test indicated unequal variances (*F* = 14.8, *p* < 0.001), therefore the degrees of freedom were adjusted from 417 to 336. The males’ group showed higher mean (*M* = 3.25, SD = 0.987) compared to the females’ group (*M* = 2.68, SD = 1.26).

#### Gender Differences on the Multitasking in Driving While Using the Phone: Multi-Group Analysis

Based on the theoretical assumptions presented in the introduction, the effect of gender on the Multitasking in driving while using the phone was assessed using a MGSEM approach: this is extensively different from modeling gender groups within the variables included in the general structural model. In this sense, the data were split into two groups (Group 1: female; Group 2: male), presenting an acceptable sample size and optimal conditions for comparability. Using the AMOS multi-group comparison analysis, the hypothesized structural model was adjusted following a multi-group invariance-testing strategy. We also estimated the bivariate correlation analysis differentiated by gender (Table 3).

**TABLE 3:** Pearson bivariate correlations of the investigated variables.

	Variables	Bivariate correlations (2-tailed) Young Female driver								
		1	2	3	4	5	6	7	8	9
1	Carelessness	1	0.284**	−0.364**	0.298**	−0.304**	−0.226**	0.305**	0.357**	0.554**
2	Multitasking Preference (Polychronicity)	0.312**	1	−0.360**	0.230**	–0.12	–0.117	0.291**	0.305**	0.255**
3	Concentration	−0.328**	−0.308**	1	−0.268**	0.150*	0.161*	−0.154*	−0.322**	−0.353**
4	Perceived self-efficacy in Multitasking	0.343**	0.294**	−0.369**	1	−0.160*	−0.200**	0.237**	0.340**	0.530**
5	Risk perceptions when using a mobile phone while driving	−0.158*	–0.095	0.224**	–0.008	1	0.523**	−0.268**	−0.480**	−0.274**
6	Risk perception about multitasking without using a mobile phone	–0.055	–0.059	0.164*	–0.103	0.453**	1	−0.171*	−0.409**	−0.296**
7	Sensation-Seeking	0.187**	0.233**	−0.180**	0.277**	–0.058	−0.148*	1	0.341**	0.329**
8	Multitasking in driving using the phone	0.259**	0.267**	−0.278**	0.214**	−0.289**	−0.142*	0.358**	1	0.457**
9	General Multitasking Behavior	0.415**	0.301**	−0.342**	0.617**	–0.055	0.006	0.332**	0.268**	1

		**Bivariate correlations (2-tailed) Young Male driver**								

		**1**	**2**	**3**	**4**	**5**	**6**	**7**	**8**	**9**

Females = 238 Males = 186. *p < 0.05. **p < 0.01.

**FIGURE 3:**
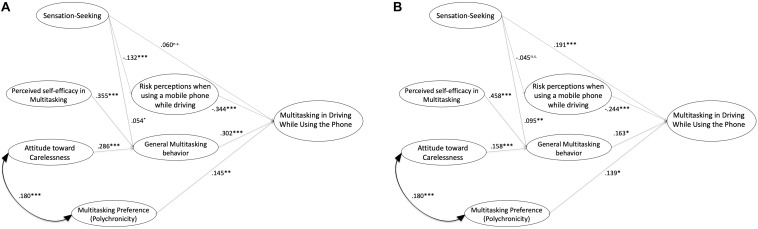
**(A)** Standardized path coefficients of structural model (MGSEM) in young female drivers. ^∗^*p* < 0.05, ^∗∗∗^*p* < 0.001. **(B)** Standardized path coefficients of structural model (MGSEM) in young male drivers. ^∗^*p* < 0.05, ^∗∗^*p* < 0.01, ^∗∗∗^*p* < 0.001.

The resulting SEM reported better fit coefficients [χ^2^ (14) = 26.238, *p* < 0.05; NFI = 0.956; CFI = 0.978; RMSEA = 0.046; CMIN/DF = 1.874) and is presented in Table 4 and Figures 3A,B. In addition to the multi-group invariance test, indicating that the model works similarly well for both of them, the RMSEA (<0.08), NFI/CFI (>0.90) coefficients suggested an optimal fit for the final model (Hu and Bentler, 1999; Yuan and Chan, 2016), showing that factor loadings, intercepts and residual covariances, were operating equivalently in both groups.

**TABLE 4:** Gender-based Multi-Group (MGSEM) model for predicting the multitasking in driving using the phone.

Dependent Variable		Independent Variable	Std. Estimate^a^	SE^b^	CR^c^	*p*	Std. Estimate^a^	SE^b^	CR^c^	*p*
				
			Young Female drivers				Young Male drivers			
Multitasking in driving using the phone	←	Multitasking Preference (Polychronicity)	0.145	0.054	2.714	0.007	0.139	0.059	2.342	0.019
Multitasking in driving using the phone	←	Risk perceptions when using a mobile phone while driving	−0.344	0.059	−5.859	0.001	−0.244	0.054	−4.553	0.001
Multitasking in driving using the phone	←	General Multitasking Behavior	0.302	0.064	4.680	0.001	0.163	0.073	2.239	0.025
Multitasking in driving using the phone	←	Sensation-Seeking	0.060	0.032	1.890	0.059	0.191	0.044	4.300	0.001
General Multitasking Behavior	←	Attitude toward Carelessness	0.286	0.042	6.809	0.001	0.158	0.039	4.063	0.001
General Multitasking Behavior	←	Sensation-Seeking	0.054	0.027	1.981	0.048	0.095	0.031	−3.013	0.003
General Multitasking Behavior	←	Perceived self-efficacy in Multitasking	0.355	0.054	6.610	0.001	0.458	0.049	9.318	0.001
Risk perceptions when using a mobile phone while driving	←	Sensation-Seeking	−0.132	0.036	−3.665	0.001	−0.045	0.050	−0.889	0.374

*^a^SPC, Standardized Path Coefficients (can be interpreted as linear regression weights). ^b^SE, Standard Error. ^c^CR, Critical Ratio.*

The MGSEM model shows that both groups keep similar characteristics regarding the use of mobile phone while driving. This is further evidence that our model appears to be adequate.

The most evident gender difference relates to sensation seeking. In young male drivers, the sensation-seeking variable has a significant effect on mobile phone use while driving (β = 0.191 ^∗∗∗^), whereas in young female drivers it is not significant (β = 0.060). Moreover, the sensation-seeking variable is not significant in young male drivers with regard to risk perception (β = 0.045), whereas it turns to be significant in young female drivers (β = −0.132^∗∗∗^).

## Discussion and Conclusion

The primary aim of this study was to assess which variables influence the use of mobile phones while driving among young adults. Findings showed a simple, yet consistent SEM model that describes how mobile phone use among young adults is predicted by some variables.

The results showed that attitudes, particularly the variable Attitude to multitask, play an important role in our model; this seems to be in line with a substantial literature showing that behavior is strongly influenced by personal attitudes (Fishbein and Ajzen, 1975; Ajzen and Albarracín, 2007; Belleau et al., 2007; Jaccard, 2012). The value (positive or negative) that individuals attribute to a particular behavior affects the engagement in risky behaviors (Neighbors et al., 2013), also in the specific case of road behavior (Iversen and Rundmo, 2002; Dahlen et al., 2005). In our case, we assessed attitudes towards concentration, carelessness and preference for multitasking (Polychronicity).

Carelessness and Polychronicity seem to be positively related; only the variable positive attitude towards concentration does not seem to predict mobile phone use while driving, nor general multitasking behavior. In addition, within the model we considered self-efficacy in multitasking (perceived behavioral control), which refers to the subject’s perception of their ability to perform a certain behavior. The theory of planned behavior suggests a relevant role of perceived control with respect to behavioral intentions (Ajzen, 1991; Madden et al., 1992). In our research, the perception of being able to control the execution of simultaneous actions has shown to be positively related to multitasking behavior.

Therefore, we considered risk perception: several studies have shown how road risk perception influences engagement in risky behaviors (Cordellieri et al., 2019; Piccardi et al., 2021), indeed, our results appear to support the hypothesis that the variable perception of risk in driving mobile phone use, influences the variable multitasking.

Numerous studies have highlighted how risky road behaviors can be influenced by sensation seeking (Jonah, 1997; Lucidi et al., 2019); particularly among youth. This personality trait has important implications in engaging in risky behaviors; also in our model, the predisposition to seek strong sensations is shown to be positively related to mobile phone use while driving as suggested by the risk perception literature.

A last variable examined within our model is the propensity to multitask behavior, which has been shown to be a significant predictor for mobile phone use. Individuals who regularly engage in multitasking behavior are more likely to use a mobile phone while driving.

In our opinion, the model derived from our investigation has theoretical consistency, is sufficiently supported by the research data, and most importantly has significant implications for youth training activities, which will be discussed below.

In addition, exclusion of the variable “Risk perception about multitasking without using a mobile phone” is equally interesting; this variable is related to risk perception, which was still associated with multitasking but without using a phone, for instance, eating a sandwich, operating the radio, etc. This variable had not proven to be a good predictor of multitasking behavior while using the phone. A simple comparison with a *t*-test between risk perception averages showed a statistically significant difference. Multitasking involving the use of a mobile phone while driving was perceived as a considerably more dangerous behavior than other forms of multitasking. It can be assumed that multitasking related to mobile phone use has different psychological determinants than other forms of multitasking (eating, using satellite navigator, chatting in the car, etc.). Young people seem to understand the risks of using mobile phones while driving, while having a reduced risk perception compared to other forms of simultaneous behaviors. Although young people are aware of the risks, they cannot avoid using their mobile phones in the car. At the same time, they are not truly aware of the risks when conversing in the car with a passenger, smoking a cigarette or setting up a navigator (items in our survey). For example, they are unaware that even being distracted for only a few seconds by the navigator’s setting means completely losing the field of vision on the road.

Further research should clearly substantiate this hypothesis. If this hypothesis were to be confirmed, it would have important operational implications in terms of training activities or road safety education, which will be discussed below in this paper.

A second aim of this research was to assess gender differences for the considered variables.

Analysis of variance revealed a greater inclination among women to have a positive attitude toward concentration and perceived risk when driving with different secondary tasks with or without the use of a mobile phone. Males showed higher average scores in positive attitude toward Polychronicity and Carelessness, Perception of Self-Efficacy in Multitasking while Driving and Sensation Seeking. Overall, a different risk profile between female and male adolescents was confirmed, with males having a higher risk propensity (Cordellieri et al., 2016, 2019). Males’ greater propensity to engage in risky road behavior also seems to be encouraged by gender stereotypes (Yeung and von Hippel, 2008; Chateignier et al., 2011; Moè et al., 2015; Pravossoudovitch et al., 2015).

Our results showed that young male drivers are more likely to use their mobile phones while driving. An interesting aspect is that there are no gender differences for general multitasking (i.e., studying while listening to music). This finding is in an agreement with several studies showing that there are no gender differences in multitasking (Hirsch et al., 2019). Hirsh and colleagues showed that beliefs that women are more likely to multitask are due to stereotypes. It is possible that the difference between general multitasking and multitasking with mobile phone use while driving must be considered within a road risk model, where males appear to be more likely to engage in risky driving behaviors than females.

Using multigroup analysis (MGSEM), the model we developed appears to be suitable for explaining the behaviors of both young male and female drivers.

Sensation seeking appears to be the only variable that differs between genders. Notably, in young female drivers this is not a significant predictor of mobile phone use while driving. We speculate that this is because the sensation-seeking variable is more present in males than in females, as has been well demonstrated in the scientific literature (Adan et al., 2016; Navas et al., 2019).

### Research Implications

This study could have important practical implications. It has been shown that risk perception is a valuable predictor of engagement in secondary tasks while driving. It was also found that risk perception differs when referring to a secondary task in driving (such as talking to a passenger, eating, other) or, in particular, use of a mobile phone. Risk-taking behaviors are therefore based on different motivations: in the first case (generic actions), they are caused by a lack of information that young drivers may have; in the second case (using the phone while driving), although they are aware of the risks involved, they cannot avoid it. These results should encourage all those working in road safety education or training to adopt a different approach in their work with young people, especially concerning the risks of secondary tasks while driving. As it is a matter of working on the risks of secondary tasks, such as distracting oneself to setting the navigator, discuss etc., an informative approach may be adequate: young people are unaware, and giving information leads to consciousness. Other than that, if the emphasis is on mobile phone use while driving, the problem cannot be approached only in terms of information: teenagers are already aware of this issue. In this case, it is appropriate to work on attitudes, deep beliefs and self-regulatory processes underpinning risky behaviors, such as using a mobile phone while driving (Giannini et al., 2013).

### Research Limitations

The results of this study need to be interpreted in light of some limitations. Firstly, we used self-reported driving behavior, which may have been affected by social desirability or recall biases, undermining the reliability of the study. Although all the necessary checks were made in the database, excluding all outliers, and in the processing of the statistical analyses, some risks cannot be completely avoided. However, the fact that the questionnaires were answered anonymously, decreased this risk (Lajunen and Summala, 2003). Also, during the data analysis, a few evidently acquiescent responses were excluded. Future studies that use more objective measures of driving behavior, such as driving simulators and/or external evaluation of road driving, are needed.

We are aware that some of our interpretive assumptions should be verified by additional research. Furthermore, other studies could test alternative models to ours, using different and/or additional variables. For example, we selected five items from the “NEO Personality Inventory” (Costa and MacCrae, 1992) to measure sensation seeking. In our research, we considered only the seeking for excitement. The Zuckerman Sensation Seeking Scale (SSS-V, 1964) includes four different aspects: Thrill and Adventure Seeking (TAS); Disinhibition (Dis); Experience Seeking (ES); and Boredom Susceptibility (BS). Boredom Susceptibility, or the other sensation-seeking variables, could also have a significant relationship with mobile phone use while driving. Therefore, in future research, it is necessary to test variables not considered by us.

A final limitation is the sample size, which does not allow us to consider our results representative of the population of young drivers. Future studies aiming to replicate our study in different samples are also needed in order to provide additional evidence for the generalizability of our conclusions.

## Data Availability Statement

The raw data supporting the conclusions of this article will be made available by the authors, without undue reservation.

## Ethics Statement

The studies involving human participants were reviewed and approved by The study was approved by the Ethics Review Board of the Department of Social and Developmental Psychology, “La Sapienza” University of Rome. Written informed consent to participate in this study was provided by the participants’ legal guardian/next of kin.

## Author Contributions

AF and PC: realization of questionnaires. MB, AP, GL, EM, and JB: administration of questionnaires. MB, AP, GL, EM, JB, and EP: data base construction and data entry. PC, AF, and AQ: data analysis. PC, AF, AQ, MB, AP, GL, EM, JB, EP, and AG: manuscript writing. AG: supervision of all work. All authors contributed to the article and approved the submitted version.

## Conflict of Interest

The authors declare that the research was conducted in the absence of any commercial or financial relationships that could be construed as a potential conflict of interest.

## Publisher’s Note

All claims expressed in this article are solely those of the authors and do not necessarily represent those of their affiliated organizations, or those of the publisher, the editors and the reviewers. Any product that may be evaluated in this article, or claim that may be made by its manufacturer, is not guaranteed or endorsed by the publisher.
